# Association between CYP1A1 rs4646903 T > C genetic variations and male infertility risk: A meta-analysis: Erratum

**DOI:** 10.1097/MD.0000000000017084

**Published:** 2019-08-30

In the article, “Association between CYP1A1 rs4646903 T > C genetic variations and male infertility risk: A meta-analysis”,^[1]^ which appears in Volume 98, Issue 31 of *Medicine* Dr. DongLiang Lu's degree should appear as MD and affiliation a. Dr. Qiang Wei's degree should appear as MD.

The conflict of interest statement should appear as “DC, ZR, LD,LL PX, QZ and QW declare that they have no conflicts of interest.”

The Revman 5.3 company information should appear as Cochrane Collaboration, http://ims.cochrane.org/revman. The Stata 14.0 company information should appear as StataCorp LP, College Station, TX.

The correct Figure 1 is below:

**Figure:**
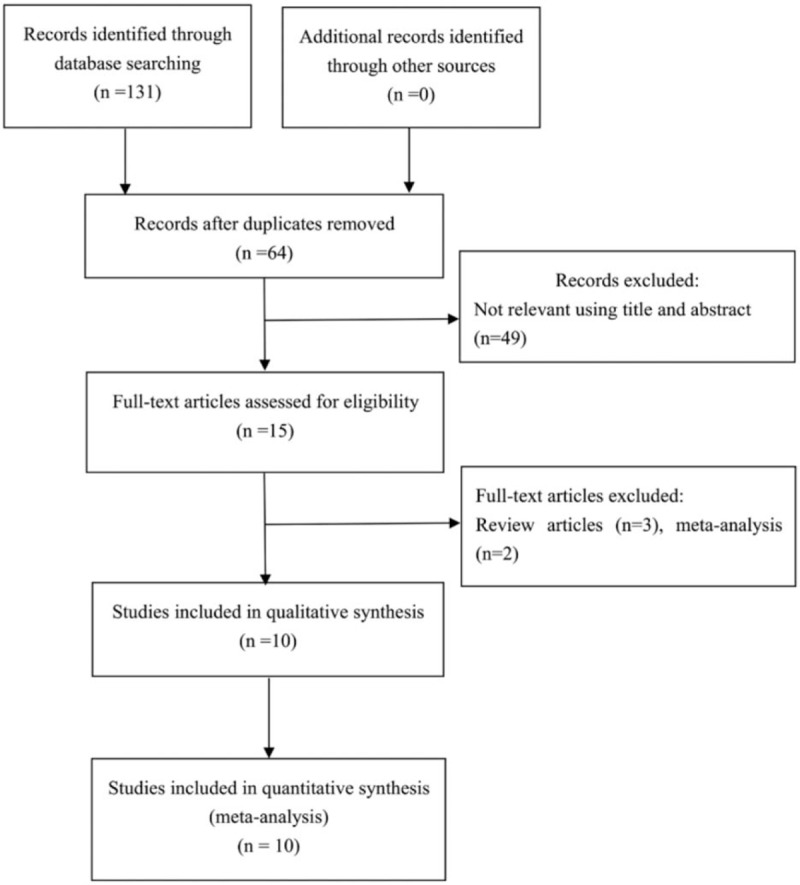


The correct Figure 2 is below:

**Figure:**
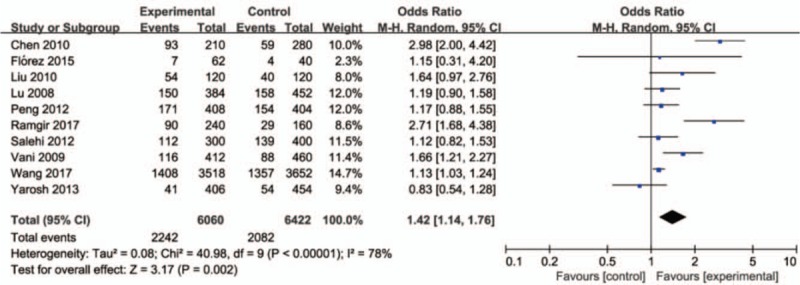


The correct Figure 3 appears below:

**Figure:**
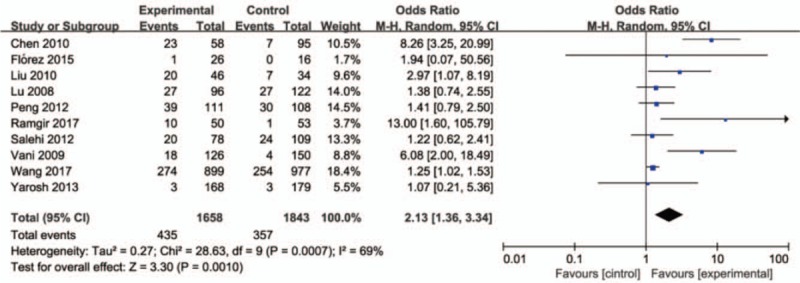


Reference 30 should also appear in this sentence: It has been suggested that the SNPs of the CYP1A1 gene could determine the activity and/or inducibility of CYP1A1.^[19,29,30]^

Reference 25 should appear as Liu B, Wang X. Genetic Susceptibility to CYP1A1 and GSTM1

Polymorphisms and Smoking With Malformed Sperm [D]. Changsha: *Central South University,* 2010.
